# Influence of peers' actual appraisals on moral self-representations of Chinese adolescents

**DOI:** 10.3389/fpsyg.2022.995206

**Published:** 2022-09-06

**Authors:** Caizhen Yue, Yihong Long, Kaihua Ou, Xiaofang Dong, Fasheng Cao

**Affiliations:** College of National Culture and Cognitive Science, Guizhou Minzu University, Guiyang, China

**Keywords:** adolescents, moral self, self-representation, reflected appraisals, peers' actual appraisals

## Abstract

Adolescence is a vital period of developing a moral self. As individuals enter adolescence, peers become increasingly important to them. This study aimed to explore the influence of peers' actual appraisals on moral self-representations. Based on Looking Glass Self Hypothesis, peers' reflected appraisals usually have a mediating effect on peers' actual appraisals and self-appraisals. This study used the *Chinese Moral Trait Words Rating Scale* to investigate 160 dyads of Chinese adolescents (12–14 years old). The participants filled in the Self-Appraisals Questionnaire, Peers' Reflected Appraisals Questionnaire, and Peers' Actual Appraisals Questionnaire, respectively. The results showed that: (a) peers' actual appraisals indirectly affected self-appraisals through peers' reflected appraisals in the process of forming the moral self of early Chinese adolescents; (b) Chinese adolescents had a certain accuracy in peers' actual appraisals, but often underestimated their peers' actual appraisals of them. This study was conducive to understanding the influence of peers on forming adolescents' moral self in the context of collectivistic culture.

## Introduction

Developmental psychologists think that as individuals enter adolescence, self-differentiation is intensified, self-content changes accordingly, and self-coherence is challenged. Therefore, searching for coherent self-awareness is an important developmental task in adolescence (Becht et al., 2016; Van Doeselaar et al., 2018). The moral self refers to the self-concept organized around a series of moral characteristics (Aquino and Reed, 2002), and as the core meaning of a person (Solomon, 1992; Blasi, 1993; Narvaez and Lapsley, 2009), it is an important part of self-knowledge. Understanding the formation of adolescent moral self helps construct adolescent self-identity, develop prosocial behavior, and shape positive personality.

Moral Self Theory (Aquino and Reed, 2002; Jennings et al., 2014) holds that the research on the moral self should focus on two aspects: the one is about the “having” of the moral self, mainly discussing how morality is internalized into an individual's self-awareness. The other is about the “doing” of the moral self, mainly discussing how internalized morality affects the cognitive and emotional self-regulation abilities that govern decision-making and behavior (Jennings et al., 2014). Researchers generally suggested that the “owning” of the moral self is both cognitive and socially constructed (Bandura, 1991; Harter, 2015). Classical symbolic interactionism thinks that people certainly would not have self-concepts if there is no interaction with others (Mead and Schubert, 1934; Swann and Bosson, 2010). Similarly, the social construction of the moral self is embedded through an individual's role, practice, and interpersonal interaction in a social moral environment, such as family, community, or organization (Hunter, 2000); its cognitive construction is generated on the basis of social interaction through the individual self-concept (Bollich et al., 2011; Srivastava, 2012), and social interaction gives meaning to their experiences. In general, when the social and cognitive constructions are based on morality, an individual is regarded as “having” moral self.

Referring to the influencing factors of the moral self, the researchers focused on personal characteristics and social factors. On the influence of personality traits on the moral self, the researchers discussed an individual's past experiences, personality characteristics, gender, etc. For example, some studies have found that the emotions of an individual's past actions and experiences have more influence on the “doing” of moral self than on the “owning” (Jordan et al., 2011); children with agreeable disposition are more likely to show concern for others (Hastings et al., 2006). In addition to individual characteristics, the researchers also explored the influence of different people, such as parents and peers, on individuals' moral self. For example, some studies have found that a supportive and warm parent-child relationship can help promote children's prosocial development (Hastings et al., 2007), while overly strict parents may undermine children's prosocial behavior; adolescents who get well along with their peers also have higher levels of prosocial behavior (Carlo et al., 2011).

How do people internalize morality as self-representations in real society? According to Looking Glass Self Hypothesis, others' actual appraisals of our moral behavior and our perceived others' actual appraisals play an important role in the formation of the moral self. Looking Glass Self Hypothesis holds that in interpersonal interactions, we are judged by others (others' actual appraisals), and we can perceive others' actual appraisals of us (others' reflected appraisals), and then we will internalize perceived others' actual appraisals into self-views (self-appraisals) (Kinch, 1963; Stets et al., 2020). The basic viewpoints of Looking Glass Self Hypothesis have been verified in different fields, for example, the influence of parents and teachers on the academic ability of middle-school students (Nurra and Pansu, 2009; Tomasetto et al., 2015), of parents, coaches, and teammates on adolescents' sports ability (Amorose, 2003; Bois et al., 2005), of parents or peers on criminal behaviors (Brownfield and Thompson, 2005; Walters, 2016), of social environments on racial identity (Khanna, 2010; Sims, 2016), of classmates in college on the teaching ability of normal university students (Hu et al., 2014), and of peers on personality formation of college students (Yue et al., 2021).

In the real world, we are in different social roles and interact with different others, and different types of others have different influences on individual self-concept. In general, if the other person is important, relevant, valued, or expected (such as a parent, or good friend), that person's perceptions or appraisals of an individual are more likely to be internalized into self-representations (Sinclair et al., 2005; Wallace and Tice, 2012). When individuals enter adolescence, adolescents take significantly less time with their parents but more time with their peers (Jankowski et al., 2014) and become more sensitive to peer acceptance or rejection of information (Pfeifer and Peake, 2012). Teenagers increasingly challenge the legitimacy of parental control over matters, such as personal appearance and cleaning the house (Smetana, 2005), and tend not to disclose to their parents what they consider to be a personal domain (Smetana et al., 2009). This means that peers are becoming increasingly important and have a growing influence on self-representations (Borghuis et al., 2017; Luan and Bleidorn, 2019; Crone and Fuligni, 2020).

From the perspective of individual development, adolescents have not yet formed a stable self-concept, and the main development task at this stage is identity exploration (Erikson, 1963; Veroude et al., 2014). With the development of individual cognitive ability and the change in the living environment in adolescence, adolescents have formed increasingly abstract self-concepts (Harter, 2015), and their self-representations have also attached growing importance to interpersonal or social characteristics (Lu, 1990). Meanwhile, adolescence is an important period of urgent integration between the individual self and morality (Damon, 1996; Blasi, 2004). For example, studies on adolescents' moral judgments have found a closer relationship between moral emotions self-attribution, and confidence in moral judgments (Krettenauer and Eichler, 2006). In addition, adolescents can reconcile their own and others' views in dealing with conflicting value information, allowing more mixed and nuanced moral appraisals, which suggested that the ability to consider their own and others' views, intentions, and emotions help individuals develop their understanding of moral experience (Wainryb et al., 2005).

As an individual meaning system constructed by individuals in social interactions (Valsiner, 2000), culture naturally affects people's moral life in a specific cultural environment. The researchers usually divide culture into two cultural types: individualism and collectivism. According to the basic view of cultural psychology, culture with an individualistic orientation (such as western societies) constructs social experience around autonomous people, is relatively detached from their communities and relationships, and is motivated to achieve freedom and personal goals. Culture with collectivism as its core (such as eastern Asian societies) constructs social experience around collectivities such as families and communities. Thus, individuals in the collectivistic culture are defined to a large extent by their interdependent roles and responsibilities prescribed by social institutions (Kitayatna et al., 2007; Triandis, 2007). Individualism and collectivism are also considered to maintain different moral concepts (Miller, 2007). Morality in individualistic culture pays more attention to individual independence and choice; while collectivistic culture is regarded as an interdependent and responsive-based morality, which mainly refers to the expectations, regulations, and responsibilities arising from individuals' roles in the social system. Previous studies have found that individuals under collectivism have higher levels of cooperation and prosociety compared with the individualistic society (Mosier and Rogoff, 2003).

The Chinese culture, which is characterized by collectivism, places more emphasis on human morality, human obligations, fulfilling social roles, maintaining social orders, and promoting social harmony. For example, some studies have found that compared with American adolescents, Chinese adolescents are more inclined to lie due to humble service, to divert their attention from individuals, and promote harmonious groups relations (Genyue et al., 2011); on children's blue lies (unconscious lies), children aged 7–11 years were presented with moral dilemmas, in which the characters either lied to help their partners or told the truth to help their community (such as school), or vice versa. Chinese children think that lies help the groups but hurt the individual less than the opposite; in addition, they suggested that telling the truth is helpful for individuals, but is harmful to the groups, so they would rather say the opposite answers (Fu et al., 2010). Based on this, we adopted the *Chinese Moral Trait Words Appraisal Scale* in line with the Chinese cultural model for research (Guo et al., 2011). According to the Chinese cultural characteristics, the scale consists of seven dimensions, namely, sympathy, agreeableness, independence, conscientiousness, extraversion, sophistication, and ambition. Sympathy refers to an individual's attitude of likes and dislikes toward others and recognition of their own beliefs in the moral context. Agreeableness refers to an individual's cooperation, selflessness, gentleness, comity, and tolerance in interpersonal communication. Independence refers to the will trait of moral behavior and is the behavior response that individuals may make in the moral context, such as strength, capability, and ratio. Conscientiousness refers to people's traits in their lifestyle and work, such as self-discipline, concentration, and seriousness. Extraversion refers to the traits of being active and positive in an interpersonal context. Sophistication refers to the basic attitudes and behavior traits of individuals to safeguard interests or achieve goals when they face complex contexts of justice and interest in society. Ambition refers to an individual's attitude and motivation toward authority and tradition.

Culture affects not only people's moral lives but also their self-representations and interpersonal interaction. The individualistic culture emphasizes the independence and uniqueness of individuals, while the collectivistic culture attaches more importance to interpersonal relations and interdependence (Triandis, 1995), thus forming independent and interdependent self-constructs, respectively (Markus and Kitayama, 1991). For individuals with individualistic tendencies, their self-representations are more likely to be constructed in a general way, while for those with collectivistic tendencies, their self-representations are more likely in a context way (Zhou and Cacioppo, 2010). Compared with American participants, Chinese participants were better at perspective-taking (Wu and Keysar, 2007); others' views often become the default position of east Asians' self (Suh, 2007). These studies meant that others' views had a greater influence on individual self-concept in a collectivistic culture.

Based on the above, this study took Chinese adolescents as the participants and focused on the domain of moral self that has core significance for individuals. Due to the increasing importance of peers in adolescence and the relatively unstable self-representations of adolescents, this study aims to explore the influence of peers on the moral self-representations of Chinese adolescents. According to Looking Glass Self Hypothesis, it is predicted that peers' actual appraisals indirectly affect adolescents' moral self-representations through peers' reflected appraisals, namely, peers' reflected appraisals have a mediating effect between peers' actual appraisals and adolescents' self- appraisals.

## Methods

### Participants

This study adopted the convenient sampling method to select a middle school in Guizhou Province. After obtaining the authorization from the school, we randomly selected 167 participants from the first grade and the second grade and asked each of them to select a partner who they were familiar with in their own class. Participants were 334 middle-school students (167 dyads). During parent-teacher meetings at the school, we introduced the study to parents in detail and conducted the questionnaire research for adolescents after obtaining their guardians' written consent. Before the questionnaire, adolescents were told that the focus of this study was to understand their appraisals of themselves and their partners, their participation was voluntary, and if they would not like to participate, they have the right to refuse. It was told them that their information would only be used for research purposes and be kept confidential. Each participant filled in three types of questionnaires: Self-Appraisals Questionnaire, Peers' Reflected Appraisals Questionnaire, and Peers' Actual Appraisals Questionnaire. Since 7 participants did not complete the questionnaires, this study collected 320 (160 dyads) of valid data. A total of 320 adolescents were 12–14 years old (*M* = 12.91, *SD* = 0.49), including 164 boys and 156 girls. This study was approved by the Ethics Committee of Guizhou Minzu University.

### Measures

#### Self-representations

We used the *Chinese Moral Trait Words Rating Scale* (CMTWRS, Guo et al., 2011) to measure the moral self-representations of adolescents. The scale has been shown to be suitable for measuring moral personality in the context of Chinese culture and comprises seven factors, namely, sympathy, ambition, independence, extraversion, conscientiousness, agreeableness, and sophistication. The scale with 59 items was assessed using a 7-point Likert scale from 1 (strongly disagree) to 7 (strongly agree). In this study, the Cronbach's alpha coefficient in the Self-Appraisals Questionnaire of the CMTWRS was 0.94.

#### Peers' reflected appraisals

To measure peers' reflected appraisal of adolescents, adolescents were asked to infer to what extent their peers thought that the trait words of the CMTWRS described themselves (adolescents). The 7-point Likert scale from 1 (strongly disagree) to 7 (strongly agree) was used for assessment. According to previous studies (Hu et al., 2014; Silva et al., 2020), “I am...” was changed into “My partner thinks I am...” in the CMTWRS. In this study, the Cronbach's alpha coefficient in the Peers' Reflected Appraisals Questionnaire of the CMTWRS was 0.96.

#### Peers' actual appraisals

To measure peers' actual appraisals, adolescents' peers were asked to fill in this questionnaire and to judge to what extent the trait words of the CMTWRS described them. The 7-point Likert scale from 1 (strongly disagree) to 7 (strongly agree) was used for assessment. “I am …” was changed into “My partner is …” in the CMTWRS. In this study, the Cronbach's alpha coefficient in the Peers' Actual Appraisals Questionnaire of the CMTWRS was 0.95.

#### IOS scale

To measure the closeness between participants and their peers, the participants were asked to fill in the “Inclusion of Other in the Self” (IOS) Scale (Aron et al., 1991). IOS was used to measure the degree of interpersonal closeness. The IOS scale consists of seven pairs of overlapping circles, with each pair overlapping slightly more than the preceding pair. The participants were asked to select the pair of circles that best portrays their relationship with their partners.

### Procedures

A total of 160 dyads of adolescents participated in this study (conducted from October to November 2018), and each dyad was composed of two participants who were familiar with each other well (the IOS score filled by 160 dyads of participants was: *M*_*self*_ = 4.93, *SD* = 1.92; *M*_*peers*_ = 5.00, *SD* = 1.84; the correlation coefficient of the IOS score filled by self-peer *r* = 0.56).

Two participants of each dyad came together to the lab, where each participant sat at a desk with a partition and two desks kept at a distance. The way was to prevent the two participants from communicating with each other while filling in the questionnaires. Each participant first filled in the Self-Appraisal Questionnaire.

Second, the participants filled in the Peers' Reflected Appraisal Questionnaire. Before filling in the questionnaire, they were asked to write their own names on the questionnaire and the names of their partners. Then, they filled in the IOS Questionnaire and were reminded what they filled in was the close relationship with the partner, and asked them to judge how the partner viewed them.

Finally, the participants filled in the Peers' Actual Appraisals Questionnaire. Before filling in the questionnaire, they were asked to write their own names on the questionnaire and the names of their partners and were reminded that the next step was to make trait words judgment on the partner.

Each dyad of participants filled in the three types of questionnaires. The Self-Appraisals Questionnaire, Peers' Reflected Appraisals Questionnaire, and Peers' Actual Appraisals Questionnaire of each dyad of participants were conducted with one-to-one correspondence. This study received 320 sets of data.

### Data analyses

The SPSS 23.0 software was adopted to process and analyze 320 sets of data, calculating descriptive statistics (*M, SD*) and Pearson correlation. Subsequently, the SPSS macro PROCESS Model 4 (Hayes and Preacher, 2013) was adopted for the mediation analyses, taking self-appraisals as the independent variable, peers' reflected appraisals as the mediator, and the peers' actual appraisals as the outcome variable; bootstrap = 5,000 resampling and 95% bias-corrected confidence intervals (*CIs*) were used. Mediation was deemed to be statistically significant if the *CIs* did not include zero.

## Results

### The test of common method variance

It is considered a thorny issue that tests the data collected from a single source and whether there are differences in common method variance (Avolio et al., 1991). This study used Harman's one-factor test (Podsakoff and Organ, 1986) to analyze common method bias (Livingstone et al., 1997). The underlying assumption of this technique is if there is a large quantity of method variations, a single factor will be isolated in factor analysis, or a common factor will be used to account for most of the variations (Fuller et al., 2016). A total of 38 factors with unrotated eigenvalues in this study were greater than 1, and the explanatory rate of variation of the first factor was 22.47%, which was lower than the critical standard of 40%, and then the results showed that the common method bias was not significant.

### Comparison of differences among self-appraisals, peers' reflected appraisals, and peers' actual appraisals

To compare the differences among self-appraisals (SAs), peers' reflected appraisals (RAs), and peers' actual appraisals (AAs), this study calculated the differences between the two types of appraisals (e.g., reflected-minus-actual) and conducted one-sample *t*-test for the difference value, with the results shown in Table 1. The results showed that RAs were significantly lower than AA in sympathy, independence, extraversion, conscientiousness, agreeableness, and moral total score; SAs were significantly lower than AAs in ambition, independence, extraversion, conscientiousness, agreeableness, and moral total score. In general, the results indicated that Chinese adolescents would underestimate peers' moral appraisals of themselves.

**Table 1:** Descriptive statistics among the three types of appraisals.

	**SA**	**RA**	**AA**	**RA-SA**	***t*_(RA−SA)_**	**AA-SA**	***t*__(_AA−SA)_**	**AA-RA**	***t*__(_AA−RA)_**
	***M(SD)***	***M(SD)***	***M(SD)***	***M(SD)***		***M(SD)***		***M(SD)***	
Sympathy	5.28 (0.93)	5.23 (1.10)	5.37 (1.08)	−0.05 (0.69)	−1.18	0.09 (1.25)	1.25	0.13 (1.32)	1.81^y^
Ambition	4.01 (1.17)	3.61 (1.28)	3.72 (1.28)	−0.40 (1.09)	−6.58^***^	−0.30 (1.61)	−3.30^***^	0.10 (1.68)	1.11
Independence	4.76 (0.99)	4.83 (1.10)	5.08 (1.07)	0.08 (0.85)	1.60	0.32 (1.28)	4.50^***^	0.25 (1.37)	3.21^***^
Extraversion	5.19 (0.96)	5.18 (0.96)	5.40 (1.00)	−0.01 (0.76)	−0.18	0.20 (1.21)	3.04^***^	0.21 (1.16)	3.29^***^
Conscientiousness	5.15 (1.03)	5.19 (1.16)	5.35 (1.20)	0.04 (0.82)	0.80	0.20 (1.41)	2.57^*^	0.17 (1.48)	2.00^*^
Agreeableness	5.00 (0.88)	5.00 (1.02)	5.12 (1.08)	−0.00 (0.71)	−0.04	0.12 (1.20)	1.83^y^	0.12 (1.26)	1.77^y^
Sophistication	2.95 (0.95)	2.95 (1.11)	3.04 (1.17)	0.00 (0.97)	0.03	0.09 (1.44)	1.11	0.09 (1.50)	1.05
Total score	4.62 (0.70)	4.57 (0.79)	4.73 (0.73)	−0.05 (0.55)	−1.58	0.10 (0.88)	2.12^*^	0.15 (0.93)	2.96^***^

SA, self-appraisals; AA, peers' actual appraisals; RA, peers' reflected appraisals; RA-SA, reflected-minus-self; AA-SA, actual-minus-self; AA-RA, actual-minus-reflected;

y p < 0.1,

* p < 0.05,

*** p < 0.001.

### Correlation analyses among self-appraisals, peers' reflected appraisals, and peers' actual appraisals

The results of correlation analyses among adolescents' moral self-appraisals, peers' reflected appraisals, and peers' actual appraisals are shown in Table 2. The results indicated that SA-RA had high correlations (*r* = 0.57–0.78) in sympathy, ambition, independence, extraversion, conscientiousness, agreeableness, sophistication, and the total score. In addition, AA-RA had low correlations (*r* = 0.14–0.28) in sympathy, ambition, independence, extraversion, conscientiousness, agreeableness, sophistication, and the total score; SA-AA had low correlations (*r* = 0.14–0.26) in sympathy, ambition, independence, extraversion, conscientiousness, agreeableness, and the total score.

**Table 2:** Correlations among the three types of appraisals.

	**SA-AA**	**AA-RA**	**SA-RA**
Sympathy	0.24^**^	0.27^**^	0.78^**^
Ambition	0.14^*^	0.14^*^	0.61^**^
Independence	0.23^**^	0.21^**^	0.67^**^
Extraversion	0.25^**^	0.31^**^	0.68^**^
Conscientiousness	0.21^**^	0.21^**^	0.72^**^
Agreeableness	0.26^**^	0.28^**^	0.73^**^
Sophistication	0.09	0.14^*^	0.57^**^
Total score	0.24^**^	0.26^**^	0.73^**^

SA-AA, the relationship between SA and AA; AA-RA, the relationship between AA and RA; SA-RA, the relationship between SA and RA.

### The mediating analyses

This study adopted Model 4 of the SPSS Macro-process (Hayes and Preacher, 2013) to test the mediating effect of RA on AA and SA.

**Figure 1:**
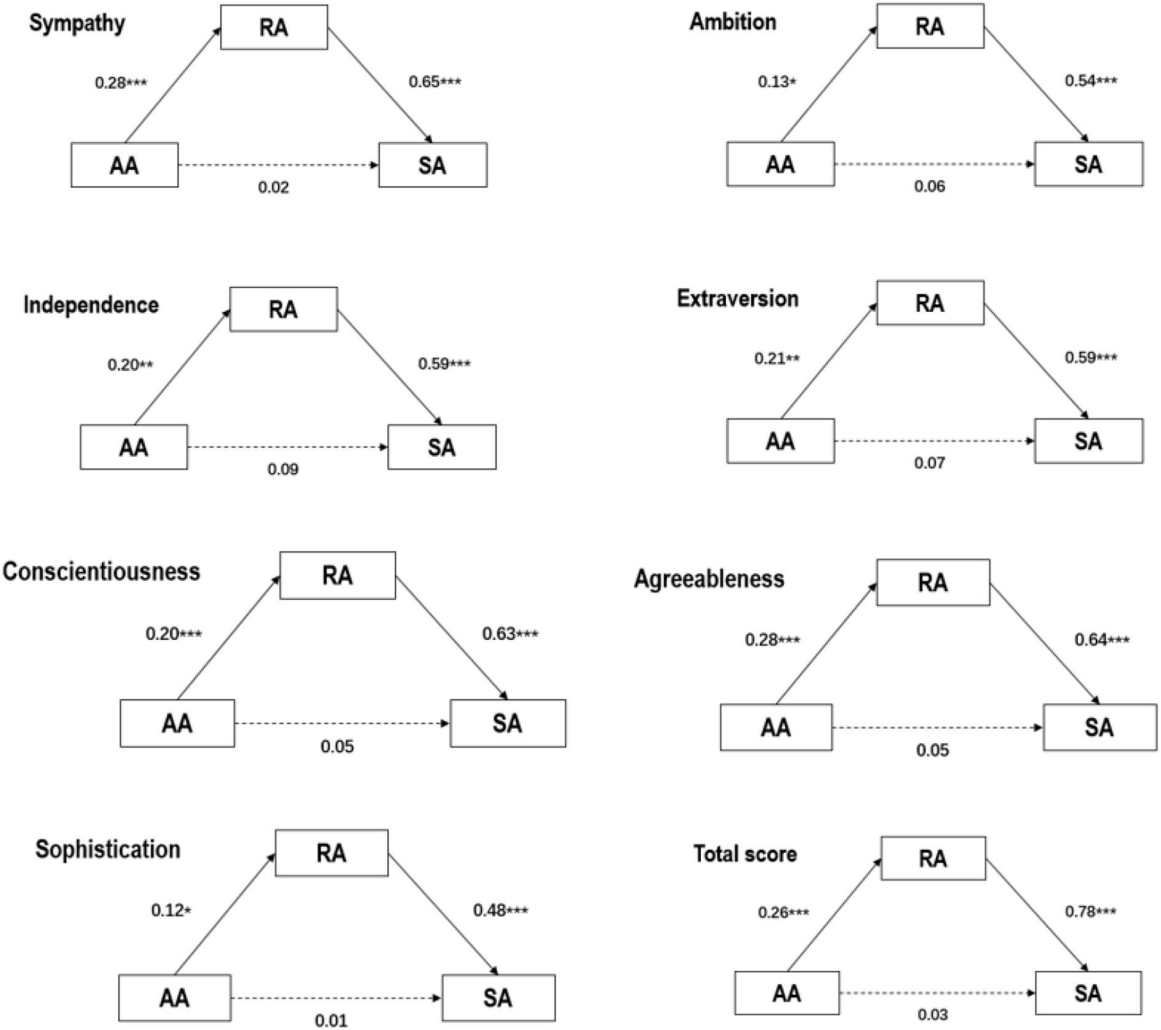
Mediation models of the effect of peers' actual appraisals and self-appraisals *via* peers' reflected appraisals.

The results showed that RA had a predictive effect on SA (sympathy, *β* = 0.65, *p* < 0.001; ambition, *β* = 0.54, *p* < 0.001; independence, *β* = 0.59, *p* < 0.001; extraversion, *β* = 0.59, *p* < 0.001; conscientiousness, *β* = 0.63, *p* < 0.001; agreeableness, *β* = 0.64, *p* < 0.001; sophistication, *β* = 0.48, *p* < 0.001; total score, *β* = 0.78, *p* < 0.001). Further analyses of bootstrap showed that RA had a significant mediating effect on AA and SA in moral personality traits (refer to Table 3; Figure 1). The mediating effects are as follows: sympathy [*ab* (mediating effect) = 0.21, *SE* (standard error) = 0.05, 95% *CI* = 0.12–0.30], ambition (*ab* = 0.08, *SE* = 0.03, 95%*CI* = 0.02–0.15), independence [*ab* = 0.14, *SE* = 0.04, 95%*CI* = 0.06–0.21], extraversion (*ab* = 0.21, *SE* = 0.04, 95%*CI* = 0.13-0.28), conscientiousness (*ab* = 0.15, *SE* = 0.04, 95%*CI* = 0.07–0.24), agreeableness (*ab* = 0.20, *SE* = 0.04, 95%*CI* = 0.12–0.28), sophistication (*ab* = 0.08, *SE* = 0.03, 95%*CI* = 0.01–0.14), and total score (*ab* = 0.20, *SE* = 0.04, 95%*CI* = 0.12–0.29).

**Table 3:** Mediating effect, direct effect, and total effect among the variables.

	**Effect**	**Boot**	**Boot**	**Boot**
		**SE**	**LL CI**	**UL CI**
**Sympathy**				
Mediating effect	0.21	0.05	0.12	0.30
Direct effect	0.02	0.03	−0.04	0.09
Total effect	0.21	0.05	0.11	0.30
**Ambition**				
Mediating effect	0.08	0.03	0.02	0.15
Direct effect	0.05	0.04	−0.03	0.14
Total effect	0.13	0.05	0.03	0.24
**Independence**				
Mediating effect	0.14	0.04	0.06	0.21
Direct effect	0.09	0.04	0.01	0.17
Total effect	0.21	0.05	0.11	0.32
**Extraversion**				
Mediating effect	0.21	0.04	0.13	0.28
Direct effect	0.04	0.04	−0.04	0.12
Total effect	0.24	0.05	0.14	0.34
**Conscientiousness**				
Mediating effect	0.15	0.04	0.07	0.24
Direct effect	0.05	0.04	−0.02	0.12
Total effect	0.18	0.05	0.09	0.27
**Agreeableness**				
Mediating effect	0.20	0.04	0.12	0.28
Direct effect	0.05	0.04	−0.02	0.13
Total effect	0.22	0.05	0.13	0.31
**Sophistication**				
Mediating effect	0.08	0.03	0.01	0.14
Direct effect	0.01	0.04	−0.07	0.08
Total effect	0.07	0.05	−0.02	0.16
**Total score**				
Mediating effect	0.20	0.04	0.12	0.29
Direct effect	0.03	0.03	−0.03	0.10
Total effect	0.19	0.04	0.10	0.28

Bootstrap sample size = 5,000; SE, standard error; LL, low limit; UL, upper limit; CI, confidence interval.

## Discussion

This study mainly discussed the influence of peers on early adolescents' moral self-representations in the context of Chinese culture. The results verified the basic view of Looking Glass Self Hypothesis and found that peers' reflected appraisals had a mediating effect on peers' actual appraisals and adolescents' moral self-perception. This study also found that there was a significant correlation between peers' reflected appraisals and peers' actual appraisals of Chinese adolescents, while the score of peers' reflected appraisals was lower than that of peers' actual appraisals, which showed that adolescents' inferences about peers' moral appraisals on themselves were accurate to a certain extent, but they often underestimated peers' actual appraisals.

This study found that peers' actual appraisals indirectly affected the moral self-representations of early through peers' reflected appraisals, which indicated that adolescent perceived peers' views were vital in the influence of others' views on their self-representations while interacting with others. This study extended the effectiveness of Looking Glass Self Hypothesis on early Chinese adolescents. The results were similar to those of previous studies taking adolescents as the participants (Silva et al., 2020; Yue et al., 2021) but different from those taking Chinese adults as the participants (Yue, 2019). Based on these research results, it was found that the influence of others' views on individuals' self-representations depended on the development stages of the individuals. This study selected early Chinese adolescents, whose self-concept was relatively unstable and whose main task was self-exploration, to form unified and complete self-representations (Van Doeselaar et al., 2018), they were more sensitive to others' feedback information (Pfeifer and Peake, 2012), and they more focused on others' appraisals of them (Harter, 2015), so others' actual appraisals had a greater effect on their self-representations. When individuals entered adulthood, their self-concept became more stable, and they moderately paid less attention to others' views whose influence on individuals' self-representations was relatively smaller (Yue, 2019). In general, both in the eastern culture and the western culture, the effectiveness of the reflected appraisals model should depend on whether the stability of the individuals' self-knowledge was or not. When individuals were in the early stage or the initial stage of new environmental adaptation (such as normal university students' early stage of their career Hu et al., 2014, they were more sensitive to the environment, and their domain self in this environment were in the stage of exploration), they would pay more attention to and infer others' views of them, and the influence of others' views on individuals' self-representations was greater. However, when individuals entered adulthood or the adjustment period in the environment, their attention to others' views was relatively lower, and the influence of others' views on individuals' self-representations was relatively smaller, for example, the studies about the accuracy of adults' reflected appraisals also found that perceived others' views were more from the self-views, not from others' actual appraisals (Carlson and Kenny, 2012).

On the relationships among self-appraisals, peers' reflected appraisals, and peers' actual appraisals, the results of this study indicated that self-reflected appraisals had high correlations, reflected-actual appraisals had medium and low correlations, self-actual appraisals had medium and low correlations, as well as the relational schemas of self-actual appraisals and reflected-actual appraisals of early adolescents were more similar. The results were different from those of researching parents' reflected appraisals of early adolescents (Silva et al., 2020), as well as different from those of researching college students (Yue et al., 2021) and adults (Yue, 2019). There were two reasons why these results were different: the one was related to the type of others. Silva et al. (2020) selected parents as important others in research and found that there were medium correlations among self-appraisals of early adolescents and parents' actual appraisals, as well as parents' reflected appraisals and parents' actual appraisals. This indicated that peers become more and more important as individuals grow up (Borghuis et al., 2017; Luan and Bleidorn, 2019), but the parent-child relationship was still the most important for individuals at the stage of early adolescents, and parents' appraisals had a greater impact on early adolescents' self-representations. The other was related to the ages of the individuals. The results of researching college students (Yue et al., 2021) and adults (Yue, 2019) found that different from the medium and low correlation between self-actual appraisals in this study, the correlation between self-actual appraisals of college students, or adults was not significant. This result could indicate that although peers' relationships were one of the most important relationships in adolescence and adulthood, peers' appraisals had a greater impact on early adolescents.

This study also found the cultural traits of peers' reflected appraisals. It was found that, in general, self-appraisals and peers' reflected appraisals were lower than peers' actual appraisals across all dimensions of moral personality, which showed that, compared with others' actual appraisals of them, Chinese adolescents appraised themselves less positively and underestimated their others' appraisals of them. This result was similar to those of previous studies on Chinese college students and adults (Hu et al., 2014; Yue, 2019; Yue et al., 2021); however, different from studies on western participants, which found that western individuals generally overestimate others' actual appraisals of them (Carlson and Kenny, 2012). The fact that Chinese individuals often underestimate how others evaluate them may reflect cultural differences in social interaction. Different from a western culture which emphasizes individual independence and individual choice, the Chinese culture puts more emphasis on interpersonal interdependence, pays attention to social order, promotes interpersonal harmony (Kim et al., 2006), and considers “face” in interpersonal interaction (Cao et al., 2009), which means others' actual appraisals on individuals will be more indirect and implicit (Hu et al., 2014), especially in moral appraisals, and others will have more positive feedbacks, but will not directly express negative information. In addition, the Chinese culture places great emphasis on individual reflection. For example, “I reflect on myself three times a day” requires individuals to have more introspection about themselves and view themselves from different angles. In addition, the Chinese culture emphasizes individuals' reflectivity more, for instance, “one should reflect on oneself in a day” requires individuals to be more introspective about themselves and to view themselves from different perspectives. The pursuit of interpersonal harmony in the Chinese culture makes people not show their uniqueness too much in group interactions but cover up their own uniqueness to make themselves harmonious with groups. The exhortation of “the tall trees that rise above the forest are always blown first by the strong wind” also causes individuals to take relatively low-key behaviors, showing lower self-appraisals and more adopting a self-modesty approach in interpersonal interactions (Hu and Huang, 2009). Therefore, based on the cognitive approach of self-modesty and the implicitness of interpersonal expression, Chinese adolescents show relatively low self-appraisals and underestimate others' appraisals of them.

This study verified the validity of Looking Glass Self Hypothesis in the collectivistic culture, but it also had some limitations. First, as the relationship between adolescents and their peers is developing gradually, how do peers' actual appraisals affect individual self-representation? In the future, longitudinal research techniques should be used to try to answer this question. Second, teachers, as significant others, have a greater impact on forming adolescents' morality. It is worth further exploring whether Looking Glass Self Hypothesis is efficient in the effect of teachers on adolescents' moral self-representations. Finally, whether the results of this study can be expanded to other groups under the collectivistic culture, the following study can be tried to expand to other countries (such as Japan and Korea) under the collectivistic culture, which can be helpful to learn more about whether individuals will underestimate others' actual appraisals under the collectivistic culture, and to understand the stability of the cultural features of others' reflected appraisals.

In conclusion, the theoretical perspectives of Looking Glass Self Hypothesis were tested under the collectivistic culture, and this study found that there was cross-cultural applicability of Looking Glass Self Hypothesis. This study showed that peers' actual appraisals indirectly affected the moral self-representations of early Chinese adolescents through peers' reflected appraisals. Compared with college students or adults, the relational schemas of self-actual appraisals and reflected-actual appraisals of early adolescents were more similar. This study also found that the cultural features of reflected appraisals, different from western participants overestimated others' appraisals of them, Chinese participants often underestimated others' actual appraisals of them. This study would be beneficial to understand the formation of Chinese adolescents' moral self-representations.

## Data availability statement

The raw data supporting the conclusions of this article will be made available by the authors, without undue reservation.

## Ethics statement

The studies involving human participants were reviewed and approved by Ethics Committee of Guizhou Minzu University. Written informed consent to participate in this study was provided by the participants' legal guardian/next of kin.

## Author contributions

CY designed the study. YL and KO carried out the survey. CY, KO, and XD analyzed the data. CY, YL, and FC drafted the initial manuscript and revised it. All authors approved the final manuscript as submitted and agreed to be accountable for all aspects of the work.

## Funding

This study was sponsored by Developmental Mechanism and Cultivational Path of Adolescents' Moral Personality supported by the Guizhou Province Philosophy and Social Science Planning Project in 2022.

## Conflict of interest

The authors declare that the research was conducted in the absence of any commercial or financial relationships that could be construed as a potential conflict of interest.

## Publisher's note

All claims expressed in this article are solely those of the authors and do not necessarily represent those of their affiliated organizations, or those of the publisher, the editors and the reviewers. Any product that may be evaluated in this article, or claim that may be made by its manufacturer, is not guaranteed or endorsed by the publisher.
